# Supplementary material for: Predicting visual function by interpreting a neuronal wiring diagram
Source: Nature. 2024 Oct 2;634(8032):113–23. doi: 10.1038/s41586-024-07953-5 (PMC11446822; doi:10.1038/s41586-024-07953-5)
Supplement: Supplementary file 6 — CRF and ERF predictions for individual TmY4 and TmY9 cells. Analogous to Supplementary Data 3, but for TmY target types. Shown are the top four monosynaptic pathways, the strongest pathway passing through each of the top ten intermediary types (ranking from Extended Data Fig. 7), and the trisynaptic pathway Tm1–TmY–Dm3–TmY (see the section entitled Prediction of spatial normalization). [file 41586_2024_7953_MOESM6_ESM.zip › DataS4/TmY9q/720575940635911416.pdf]

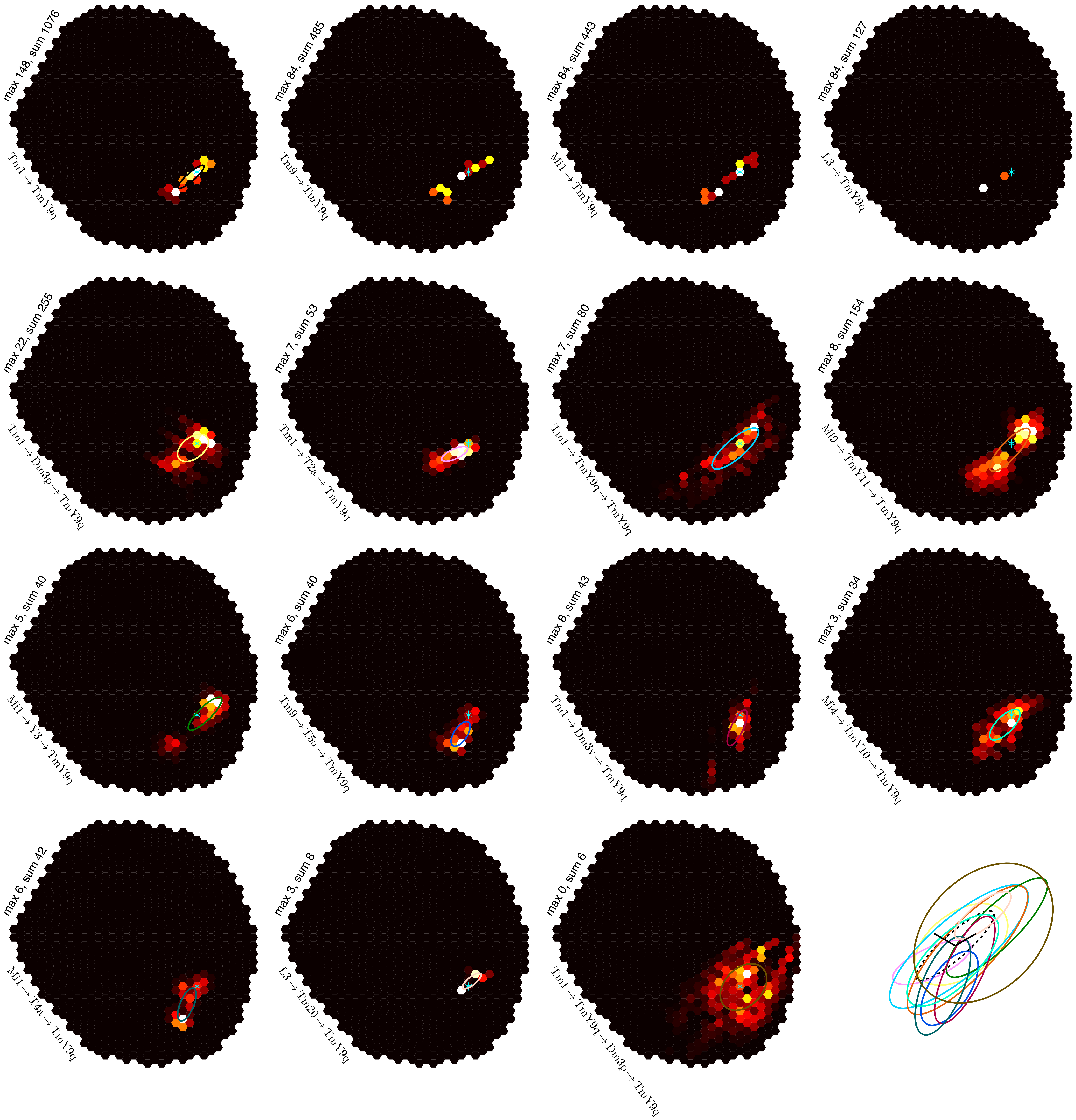
max 148, sum 1076
Tm1 → TmY9q
max 84, sum 485
Tm9 → TmY9q
max 84, sum 443
Mi1 → TmY9q
max 84, sum 127
L3 → TmY9q
max 22, sum 255
Tm1 → Dm3p → TmY9q
max 7, sum 53
Tm1 → T2a → TmY9q
max 7, sum 80
Tm1 → TmY9q → TmY9q
max 8, sum 154
Mi9 → TmY11 → TmY9q
max 5, sum 40
Mi1 → Y3 → TmY9q
max 6, sum 40
Tm9 → T5a → TmY9q
max 8, sum 43
Tm1 → Dm3v → TmY9q
max 3, sum 34
Mi4 → TmY10 → TmY9q
max 6, sum 42
Mi1 → T4a → TmY9q
max 3, sum 8
L3 → Tm20 → TmY9q
max 0, sum 6
Tm1 → TmY9q → Dm3p → TmY9q
